# Evaluation of an Antibody Detecting Point of Care Test for Diagnosis of *Taenia solium* Cysticercosis in a Zambian Rural Community: A Prospective Diagnostic Accuracy Study

**DOI:** 10.3390/diagnostics11112121

**Published:** 2021-11-15

**Authors:** Chishimba Mubanga, Inge Van Damme, Chiara Trevisan, Veronika Schmidt, Isaac K. Phiri, Gideon Zulu, John Noh, Sukwan Handali, Richard Mambo, Mwelwa Chembensofu, Maxwell Masuku, Dries Reynders, Famke Jansen, Emmanuel Bottieau, Pascal Magnussen, Andrea S. Winkler, Pierre Dorny, Kabemba E. Mwape, Sarah Gabriël

**Affiliations:** 1 Department of Clinical Studies, School of Veterinary Medicine, University of Zambia, Lusaka 10101, Zambia or Chishimba.mubanga@ugent.be (C.M.); igphiri@yahoo.co.uk (I.K.P.); gideonzulu@yahoo.com (G.Z.); richardmambo2@gmail.com (R.M.); malronc2003@yahoo.co.uk (M.C.); mmasuku69@yahoo.com (M.M.); evans.mwape@unza.zm (K.E.M.); 2 Department of Veterinary Public Health and Food Safety, Faculty of Veterinary Medicine, Ghent University, 9820 Merelbeke, Belgium; inge.vandamme@ugent.be (I.V.D.); ctrevisan@itg.be (C.T.); 3 Department of Biomedical Sciences, Institute of Tropical Medicine, 2000 Antwerp, Belgium; fjansen@itg.be (F.J.); pdorny@itg.be (P.D.); 4 Department of Neurology, Center for Global Health, Faculty of Medicine, Technical University of Munich, 81675 Munich, Germany; veronika.schmidt@tum.de (V.S.); andrea.winkler@tum.de (A.S.W.); 5 Centre for Global Health, Institute of Health and Society, Faculty of Medicine, University of Oslo, 0450 Oslo, Norway; 6 Eastern Provincial Health Office, Ministry of Health, Chipata 510023, Zambia; 7 Division of Parasitic Diseases and Malaria, Centers for Disease Control and Prevention, Atlanta, GA 30333, USA; jnoh@cdc.gov (J.N.); ahi0@cdc.gov (S.H.); 8 Department of Applied Mathematics, Computer Science and Statistics, Faculty of Sciences, Ghent University, 9000 Ghent, Belgium; dries.reynders@ugent.be; 9 Department of Clinical Sciences, Institute of Tropical Medicine, 2000 Antwerp, Belgium; EBottieau@itg.be; 10 Faculty of Health and Medical Sciences, University of Copenhagen, 2200 Copenhagen, Denmark; pma@sund.ku.dk

**Keywords:** *Taenia solium*, human cysticercosis, point of care test, diagnosis, screening, antigen ELISA, rT24H EITB, sensitivity, specificity

## Abstract

The lack of cheap, easy-to-use, rapid diagnostic tests has led to the development of several rapid diagnostic tests for cysticercosis. The new prototype two-strip, *Taenia solium* point of care test (TS POC) detects antibodies against taeniosis (TS POC T) and cysticercosis (TS POC CC). This study evaluated the diagnostic performance of the TS POC CC in the Sinda district in eastern Zambia. A sample of 1254 participants was recruited and tested with the TS POC. Out of the 1249 participants with a valid TS POC result, 177 (14%) tested positive while 1072 (86%) tested negative. All individuals with a positive TS POC and a subset of negative TS POC participants were selected for serum sampling, and were subjected to the recombinant glycoprotein T24H enzyme-linked immunoelectrotransfer blot (rT24H EITB) and the serum B60/158 (serum Ag) enzyme-linked immunosorbent assay (Ag ELISA). Performance characteristics were estimated using a Bayesian approach with probabilistic constraints. Based on 255 complete cases, the estimated sensitivity and specificity of the TS POC CC test were 35% (95% CI: 14–63%) and 87% (95% CI: 83–90%), respectively. The diagnostic performance needs to be improved, possibly by titrating antigen and other reagents’ concentration in the strip to produce a performance similar to existing cysticercosis tests such as the rT24H EITB.

## 1. Introduction

Human cysticercosis (HCC) caused by infection with *Taenia solium* is a disease of public health importance. The neural form of HCC, neurocysticercosis (NCC), is of clinical significance, as it is associated, with epilepsy, among other afflictions. HCC is epidemiologically important in determining *T. solium* community infection dynamics and its associated risk factors [1,2], initiation of control interventions [3,4,5,6], and, sometimes, in the measuring of the effectiveness of control interventions [6].

HCC diagnosis is challenging because the disease is often asymptomatic. It is mostly diagnosed serologically by antibody and antigen enzyme-linked immunosorbent assay (ELISA) and lentil-lectin glycoprotein enzyme-linked immunoelectrotransfer blot (LLGP EITB) [7]. The majority of existing HCC diagnostic tests have been evaluated and validated for NCC diagnosis based on neuro-imaging results of the brain only. LLGP EITB is very sensitive and specific and the test of choice for cysticercosis serodiagnosis (sensitivity 98%, specificity 100% [7,8,9,10]. However, due to its complex development and the need for native parasite antigen, a recombinant based EITB using the rT24H antigen has been developed (rT24H EITB) [11]. This test has only been validated for NCC diagnosis and its performance was concordant to that of the LLGP EITB [12]. However, this test is not commercially available. Several cysticercosis commercial EITBs have since been developed with varying degrees of performance. These include the Qualicode^TM^ cysticercosis western blot kit, with a sensitivity of 95% and a specificity of 100% (Immunetics, USA), and the LDBIO Western blot kit, with a sensitivity of 97.5% and a specificity of 100%. Similarly, several antibody ELISAs have been developed and commercialized. However, most perform poorly with low sensitivity and specificity [10]. On the other hand, two antigen detecting ELISAs, the B158/60 (serum Ag ELISA) [13] and the HP10 antigen ELISA [14] are available and recommended for use in diagnosis, detecting primarily the presence of viable cysticerci [15,16]. A commercial version of the B158/60 serum Ag ELISA is available as well [10]. These existing reference tests are not suitable for deployment in the community within resource-poor settings, as they are expensive, time consuming, may require sophisticated equipment and infrastructure, and require highly trained people to administer them [17].

The lack of cheap, easy-to-use, rapid diagnostic tests has led to the development of several rapid diagnostic tests for cysticercosis [18]. Despite this, none of the developed tests have been evaluated for practical use in either health facilities or communities. Recently, a new, single-cassette, bi-strip, prototype point of care test (TS POC) based on the recombinant antigens rES33 [19] and rT24H [11], for simultaneous detection of anti-taeniosis (TS POC T) and anti-cysticercosis (TS POC CC) antibodies, respectively, has been developed by the Centers for Disease Control and Prevention (CDC, Atlanta, GA, USA) collaborating with the Technical University of Munich (TUM, Munich, Germany). The objective of this study was to evaluate the TS POC CC sensitivity and specificity for diagnosis of HCC in a community setting. This evaluation was done within the scope of the “Evaluation of an antibody detecting point-of-care test for the diagnosis of *Taenia solium* taeniosis and (neuro) cysticercosis in Tanzania and Zambia” (SOLID) project, a study implemented in a hospital setting and a community setting in Tanzania and Zambia, respectively. As such, this article specifically focuses on the TS POC diagnostic performance for HCC, and is part of a series of articles covering the evaluation of the test for HCC.

## 2. Methods

The detailed methodology has been published [20]. A prospective diagnostic accuracy study was undertaken, using a two-stage design. Due to the lack of a gold standard to detect HCC, three different reference tests were performed on serum samples (EITB LLGP, rT24H EITB, and serum Ag ELISA), and the performance measures of the TS POC CC test were assessed using a Bayesian approach with probabilistic constraints.

### 2.1. Study Setting, Design and Participant Recruitment

The study setting was the Sinda district in the Eastern Province of Zambia, an area known for *T. solium* endemicity, free range pig husbandry and low sanitation practices [21]. A selection of study communities was based on previous reports of porcine cysticercosis, the availability of a local health center, community willingness to participate, and all-season accessibility. Four communities, Mtore, Butao, Chinzure and Ndaula, that are covered by the Mtandaza local clinic were selected.

To obtain a desired precision around the estimates of sensitivity and specificity of 10%, a target sample size of 1200 participants was calculated. To be eligible for the study, participants had to live in the study area, be 10 years of age or older, give written informed consent and express willingness to participate in all study aspects, such as getting tested, providing of blood and stool samples, as well as going for a computed tomography (CT) scan if and when it was required. Those who were reported severely ill, pregnant or visiting the study area were not recruited for the study.

A census was carried out before the recruitment started. The target sample size was allocated to each village proportional to its census population. In the village, participating households were randomly selected. In each household, all consenting, eligible individuals were recruited. If a household was absent/refused, the next household on the random list was visited. Recruitment was conducted according to the randomized order until the village target number was met. The recruitment took place from December 2017 to June 2019.

### 2.2. Sampling and Sample Processing

**Figure 1:**
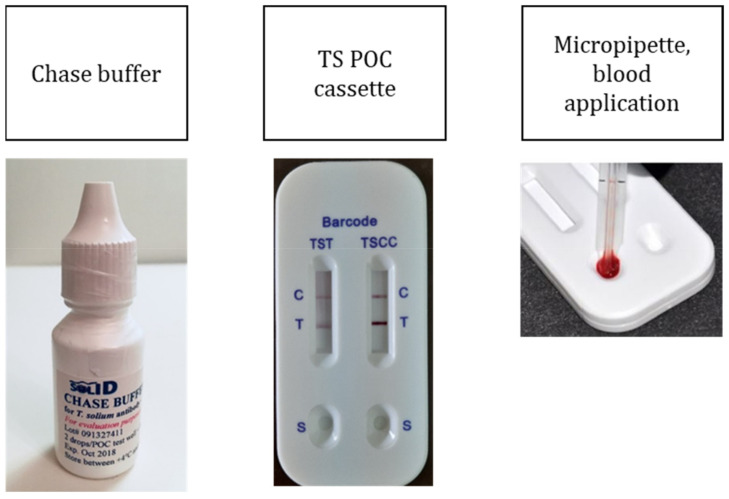
*Taenia solium* TS POC kit, chase buffer, cassette and micropipette.

All participants who tested positive on at least one of the TS POC test strips (TS POC T and/or TS POC CC, Figure 1), and a systematically selected 20% of participants who tested negative on both test strips, were requested to give a blood and stool sample. The latter sample was used for taeniosis diagnosis (processing and results reported in Mubanga et al. 2021, submitted). A trained nurse or clinician sampled 3 mL of venous blood from the participants. Blood samples were processed by allowing the tubes to stand overnight at 4 °C prior to being centrifuged at 3000 rpm for 15 min. The serum was then aliquoted into two vials and stored at −20 °C until reference testing. All samples were given a code by barcoding.

### 2.3. Diagnostic Methods

#### 2.3.1. Index Test—The TS POC CC Test Strip

The prototype TS POC is a standard lateral flow assay with two test strips in one cassette developed for diagnosis of cysticercosis (TS POC CC) and taeniosis (TS POC T) (Figure 1) [22]. It is based on two previously characterized and used recombinant proteins, rT24H [23] and rES33 [11,19]. Each test strip has a separate sample port, test and control line and an absorbent pad. Each cassette is sealed in an aluminum pouch with a desiccant and labelled with a production and expiry date as well as a batch number. The reported initial laboratory performance of the TS POC CC had a sensitivity range of 88–93% and 99% specificity while for TS POC T, sensitivity was 82% and specificity 99% (CDC/TUM unpublished).

The test was performed using finger-prick blood. Fingers were cleaned and disinfected with an ethanol swab. After pricking the lateral aspect of the chosen finger with a lancet, 20 µL whole blood was collected using a micropipette and placed in the sample port for cysticercosis. A different pipette was used to collect another volume of blood for the taeniosis port. Two drops of chase buffer were immediately added to the sample ports. When the mixture started to flow, a timer set to 20 min was started. A red line appearing at the control mark indicated a valid result, while its absence indicated a negative one. The results were read by a trained community health worker followed by the clinician or the nurse. In case of disagreement, a third person, usually the researcher, read the results. The results were recorded on the results card by the clinician or nurse and the researcher entered them into Epi Collect 5 (https://five.epicollect.net/ (last accessed on 28 August 2019)). There was no adverse event recorded during the performance of the TS POC CC or when blood samples for reference testing were drawn.

#### 2.3.2. Reference Tests

Three reference tests were used, the LLGP EITB (Se 98%, Sp 100%) [8], the rT24H EITB (Se 94%, Sp 98%) [11,23], which has a performance concordant to the LLGP EITB [12] and the B158/60 serum Ag ELISA (Se 80%, Sp 97% for infection) [9,13]. The EITB performances presented are for NCC as the tests have not been evaluated for HCC. The chosen reference tests are the recommended tests for cysticercosis diagnosis [15,16].

The LLGP EITB and the rT24H EITB were used as previously described [8,11], but with a few modifications. The dilution of the conjugate was at a ratio of 1:1000. The commercial 3, 3′-Diaminobenzidine (DAB) and the hydrogen peroxide were used according to the manufacturer’s instructions (12% instead of 10% of 30% hydrogen peroxide) (Sigma Aldrich, Steinheim am Albuch, Germany). Both the LLGP and the combined recombinant coated strips (rT24H and rES33) were obtained from CDC, Atlanta. The LLGP EITB and the rT24H EITB are both in-house diagnostic tests for the CDC. At the time of the laboratory analysis, the CDC was unable to perform these procedures due to limited manpower, and the reagents were not sufficiently available. Therefore, they shared the protocol, some of the necessary reagents (conjugate, PBS, GAHG, and DAB), and positive controls for quality control. As these reagents were only sufficient for quality control during transfer and setup of the test in the laboratory, commercial reagents were used for analysis of samples. As such, a modification in dilutions was done to produce the same quality of outcome as when the CDC reagents were used.

The serum Ag ELISA was used as previously described [13]. The serum Ag ELISA was performed at the Cysticercosis Regional Reference Laboratory, University of Zambia, while the EITBs were performed at the Institute of Tropical Medicine, Antwerp, Belgium. One researcher (CM) who was part of the recruitment in the community also took part in the laboratory analysis in Zambia. The other laboratory analysts were completely blinded to the TS POC result and the serum Ag ELISA or EITB results. The EITB tests were performed in parallel (so not blinded to the other EITB results).

### 2.4. Data Analysis

Descriptive statistics were applied for demographic characteristics of the participants, using R version 4.0.2. Sensitivity and specificity (co-primary endpoints), positive and negative predictive values (PPV, NPV), and prevalence (exploratory endpoints) were estimated using a Bayesian approach with probabilistic constraints as previously described [24,25]. The original approach was expanded by altering the multinomial probabilities according to the observed sampling frequencies to account for the TS POC CC-based sampling. Open BUGS software version 3.2.3 (www.openbugs.net (installed 15 March 2019)) was used for Bayesian analysis. Probabilistic constraints used were in the form of expert opinion obtained from three experts from the Institute of Tropical Medicine (PD), Ghent University (SG) and the University of Zambia (KEM) (Supplementary Materials File S1). Based on these prior estimates, several models were constructed with different levels of constraint (Supplementary Materials File S1). Fifty thousand iterations were run and 25,000 discarded as burn-in. After checking convergence, model fit was compared through the deviance information criterion (DIC) and the Bayesian P and pD (evaluated in both the multinomial probabilities and the model parameters as advised by Berkvens [25]). Although we initially planned to include three reference tests in the model, the LLGP EITB results had to be removed because they did not satisfy the internal quality control during the laboratory analyses (see results).

The analysis was applied using observations that had a result for the TS POC CC as well as all the reference tests that were included in the model (complete case analysis).

To explore agreements between the different tests, Cohen’s kappa statistics were calculated [26]. Since these are not good estimates for overall agreement when the number of positive tests are low, positive and negative agreements were calculated according to Cicchetti and Feinstein [27]. To calculate the point estimates of these measures for agreement, the observed frequencies of the complete cases were inversely weighted according to their sampling frequencies.

### 2.5. Trial Registration

The SOLID study was registered in the Pan African Clinical Trial Registry PACTR201712002788898.

## 3. Results

### 3.1. Flow and Demographic Characteristics of Participants

**Figure 2:**
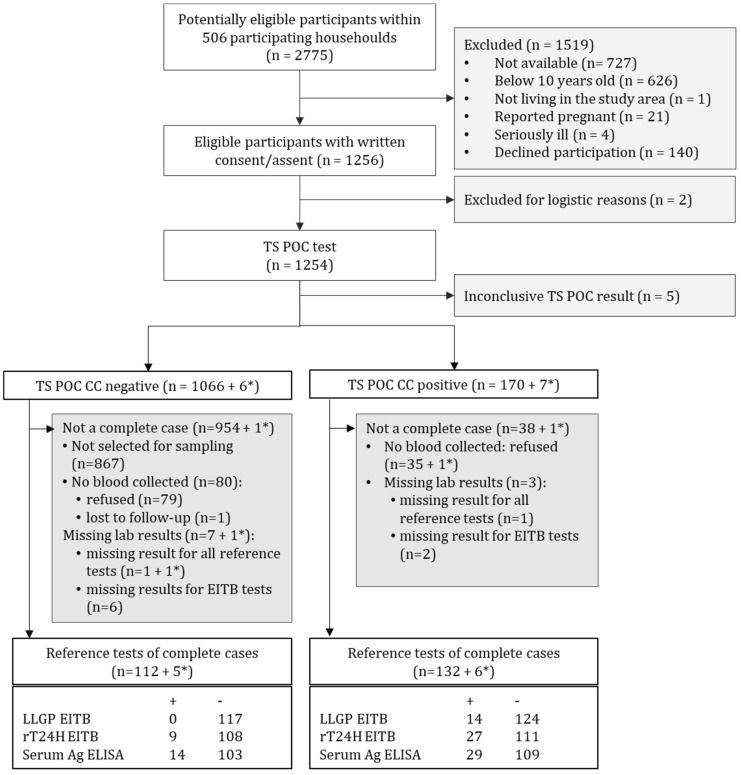
Flow diagram of participants to assess the diagnostic accuracy of the TS POC CC test strip (index test). (*) Number of participants who tested positive on the TS POC T test strip. In total, 13 participants tested positive using the TS POC T test strip, and all were selected to give a blood and stool sample.

From the census, the total population in the four communities was 4331, living in 862 households in 40 different villages. In total, 2775 people within 506 participating households were screened for eligibility, and informed consent/assent was obtained from 1256 people (Figure 2). The TS POC CC was performed on 1254 participants. The demographic characteristics of the TS POC-tested participants are shown in Table 1.

Out of 1254 participants, five had inconclusive TS POC results, leaving 1249 participants with a valid TS POC result, from which 1072 tested negative while 177 tested positive on the TS POC CC strip (Figure 2). In total, six of the TS POC CC negative and seven of the TS POC CC positive participants tested positive using the TS POC T strip. While all participants who were positive using at least one TS POC test strip were eligible for sampling, only 199 (1066–867 from the flow chart) out of 1066 participants who tested negative on both TS POC strips were selected for sampling. In total, a blood sample was collected from 125 TS POC CC negative and 141 TS POC CC positive participants (obtained by adding missing lab results and reference tests of complete cases from the chart).

Cases with results available for the TS POC CC and all the reference tests were considered as complete cases. For 11 of the blood samples (7 + 1* in the TS POC CC negative group, three in the TS POC CC positive group), one or more reference test results were missing (Figure 2). The serum Ag ELISA was the first reference test that was performed, and eight samples (negative with the serum Ag ELISA) could not be analyzed with EITB because the remaining volume was too low. In total, 117 and 138 complete cases were obtained from the TS POC CC negative and positive group, respectively, resulting in a total of 255 complete cases that were used for further analysis.

### 3.2. Reference Test Results

The result combination of complete cases (n = 255) is shown in Table 2. Using LLGP EITB, none of the serum samples were LLGP EITB positive within the TS POC CC negative group (n = 117) while 14 samples tested positive within the TS POC CC positive group (n = 138) (Figure 2). The color intensity of the positive control of the LLGP EITB using commercial reagents did not meet the internal quality control threshold (color was very faint), therefore, the quality of the sample LLGP EITB analyses could not be guaranteed. Using rT24H EITB and serum Ag ELISA, nine and 14 of the TS POC CC negative participants compared to 27 and 29 of the TS POC CC positive participants tested positive, (Figure 2).

The complete cases were used to estimate the diagnostic accuracy of the TS POC CC test by Bayesian analysis.

### 3.3. Estimates of Diagnostic Accuracy

The inclusion of LLGP EITB outcomes in the Bayesian analysis was only concordant with the prior information when the lower bound for the prevalence was lowered to zero (Supplementary Materials File S1), resulting in an estimated prevalence of 2%. This was considered implausible given the high *T. solium* endemicity of the area. Given that the results of the LLGP EITB had not met internal quality control, the models for the primary analysis were performed without the LLGP EITB results.

For the primary analysis, model 2 was selected based on DIC, Bayesian P and pD. Models using more confined priors resulted in bad fits, as expressed in Bayesian *p*-values close to 1. The analysis resulted in an estimated sensitivity and specificity of 35% (95% CI: 14–63%) and 87% (95% CI: 83–90%) for the TS POC CC test (Table 3), respectively. The estimated sensitivity of rT24H EITB was much higher (94% (95% CI: 91–98%)), as induced by the prior information. The estimates for serum Ag were very similar to the TS POC CC (Table 3.)

### 3.4. Agreement between Tests

Different statistics were calculated to examine the agreement between the different tests (Table 4). Within the TS POC CC positives (n = 138), only 27 samples were also positive using rT24H EITB. Moreover, nine samples within TS POC CC negatives were rT24H EITB positive, which resulted in a design-corrected positive agreement of only 24% between both tests.

## 4. Discussion

With an estimated sensitivity of 35% and a specificity of 87%, the TS POC CC performance fell below its laboratory performance. There are no target product profiles available for HCC yet. The closest target product profiles available are for NCC diagnosis, the minimum for screening being 80–90% sensitivity and 90–98% specificity [16,28]. The current performance of the TS POC CC is too low for the test to be used as a routine cysticercosis diagnostic or screening test, even in rural communities where cost and simplicity are more important than accuracy [16]. Given these results, the performance of the TS POC CC needs to be improved.

Nearly all antibody tests for cysticercosis are designed based on NCC reference sera and often the sensitivity is reported according to the number of cysticerci in the brain. Before the start of this study, the preliminary sensitivity of the TS POC CC test was estimated at 88% for one cysticercus and 93% for multiple brain cysticerci. Since the sensitivity estimates were based on reference sera from patients with CT-confirmed NCC, this likely contributed to the considerably lower sensitivity of the TS POC CC strip in the current study, which was evaluated for the detection of HCC, not NCC. Moreover, besides these differences in target condition (NCC vs. HCC) and population (symptomatic patients vs. community), the initial assessment was done under laboratory conditions, which differ considerably from the field settings in the current study.

The next steps towards optimization need to focus on HCC diagnosis, as the utility of this test is also in epidemiological studies and surveillance [6,29]. As such, improving sensitivity so as to detect low titer antibody in HCC reference sera can be done potentially by titration and optimization of antigen and reagent combinations to produce a diagnostic performance similar to existing reference tests such as the rT24H EITB. For specificity, special attention has to be paid to cross-reactions reported in some test formats between the rT24H with *Plasmodium falciparum* [30] and *Schistosoma mansoni* [7] besides other parasites. These would affect the test if used in sub-Saharan Africa.

This study also estimated the test characteristics of the rT24H EITB and the serum Ag ELISA for HCC diagnosis. The performance of the rT24H EITB on sensitivity and specificity was high, likely due to the effect of prior information, but with a moderate PPV. Although previous evaluations of the rT24H EITB have been for NCC diagnosis (Sensitivity 66–80% and 99%, Specificity 98% and 99%, respectively) [12,23], this performance is still comparable. The performance of the serum antigen ELISA, which detects viable cysts only, was lower than previously reported in community-based studies [9,31].

The NPVs were all high. This is expected in a population with such a low prevalence [32]. Previous studies in the area have reported similar observed prevalences of 5.8–13% using the serum Ag ELISA and higher estimates (36%) using EITB [21]. In contrast, the proportion of positive cases detected by the antigen test in this study was (slightly) higher than that detected by antibody tests (rT24H EITB and LLGP EITB). While this could mean there were more established, viable cysticercosis infections that had not yet developed antibodies, it is usually not the known epidemiological pattern of cysticercosis in a population [33]. During laboratory analysis, faint test lines were reported for the LLGP EITB even on positive control samples, possibly leading to low positive detection. This is probably the reason for a lack of fit between the data and prior information when the LLGP EITB results were included in the analysis. On the other hand, the test bands for the rT24H EITB were clearly visible. For now, it is not immediately clear why there were more positives on the antigen test than the antibody test. This phenomenon was also observed in another study in DR Congo [34].

Only a slight agreement was found according to the estimated Cohen’s kappa point estimates, not only between the TS POC CC test and each of the reference tests, but also among the different reference tests. Due to the relatively low number of positive cases, kappa may not be a good representation of agreement [27], as demonstrated by the high negative agreements among tests. The positive agreements between all of the tests were low, even among the antibody detecting tests sharing the same antigen, the TS POC CC and the rT24H EITB.

This study assessed the field performance of the TS POC test for the detection of HCC for its use in *T. solium* endemic resource-poor communities in sub–Saharan Africa, such as the highly endemic rural communities in the eastern province of Zambia that was chosen for our study. Nevertheless, the results of this study may not be generalized to communities with a different disease spectrum, such as communities with lower exposure due to better sanitation, hygiene or cooking habits, since such practices may result in less severe infections, which are harder to detect. Potential biases could occur from the high number of sampling refusals (especially on TS POC double negative participants) in case they would have a different disease spectrum compared to the participating population. Despite the limitations, the method of estimating diagnostic performance is one of the recommended methods in the absence of a “gold” standard [24,25,35]. The Bayesian probabilistic constraint model has been validated for such pathogens as *T. solium* [13] and Campylobacter [36]. It has also been utilized in many studies [9,37,38,39,40].

## 5. Conclusions

The prototype TS POC CC estimated sensitivity and PPV were too low for the test to be used for diagnosis or screening. Optimizing the antigen concentration in the test strip could sufficiently increase sensitivity to a screening threshold. Further refinement may be necessary for the test to be used for diagnosis in endemic communities.

## Figures and Tables

**Table 1:** Demographic characteristics of TS POC tested participants (n = 1254).

Characteristic	
Age (years)	
Mean	33
Range (min–max)	10–95
Gender	
Female (%)	718 (57.3%)
Male (%)	536 (42.7%)

**Table 2:** TS POC CC and reference result combinations of complete cases (n = 255).

TS POC CC	rT24H EITB	Serum Ag ELISA	n
1	1	1	8
1	1	0	3
1	0	1	1
1	0	0	2
1	1	1	5
1	1	0	11
1	0	1	15
1	0	0	93
0	1	1	1
0	1	0	8
0	0	1	13
0	0	0	95

The complete cases that were TS POC CC positive (n = 138) were sampled from 177 TS POC CC positive participants and the complete cases that were TS POC CC negative (n = 117) are a sample of 1072 TS POC CC negative participants. TS POC CC = *T. solium* point-of-care test for cysticercosis; rT24H EITB = recombinant enzyme-linked immunoelectrotransfer blot, serum Ag ELISA = serum antigen enzyme-linked immunosorbent assay. NOTE: 1 = positive and 0 = Negative.

**Table 3:** Estimates of diagnostic accuracy.

	Diagnostic Test		
Measure of Accuracy	TS POC CC	rT24H EITB	Serum Ag ELISA
Se (95% CI)	35 (14–63)	94 (91–98)	36 (15–67)
Sp (95% CI)	87 (83–90)	95 (91–98)	87 (81–92)
PPV (95% CI)	16 (8–26)	62 (40–86)	17 (7–32)
NPV (95% CI)	94 (89–98)	100 (99–100)	94 (89–98)

Bayesp = 0.5; prevalence 7% (CI: 5–12%); Se = sensitivity; Sp = specificity; PPV = positive predictive value; NPV = negative predictive value; CI = credibility interval; TS POC CC = *T. solium* point-of-care test for cysticercosis; rT24H EITB = recombinant enzyme-linked immunoelectrotransfer blot; serum Ag ELISA = serum antigen enzyme-linked immunosorbent assay.

**Table 4:** Agreement between each of the different tests that were used for the detection of cysticercosis.

	TS POC CC	rT24H EITB
TS POC CC	NA	
rT24H EITB	κ = 0.14 p_pos_ = 0.24, p_neg_ = 0.9	NA
Serum Ag ELISA	κ = 0.09 p_pos_ = 0.21, p_neg_ = 0.87	κ = 0.09 p_pos_ = 0.18, p_neg_ = 0.9

Cohen’s kappa point estimates (κ), positive agreement (p_pos_) and negative agreement (p_neg_) were calculated based on complete cases, which were inversely weighted according to their observed sampling frequencies. NA, not applicable.

## Data Availability

Data can not be shared publicly due to ethical and privacy concerns. However, data is readily available at the Institute of Tropical Medicine, Antwerp, through the Data Access Committee: https://www.itg.be/E/data-sharing-open-access, email: ITMresearchdataaccess@itg.be.

## Supplementary Materials

The following are available online at https://www.mdpi.com/article/10.3390/diagnostics11112121/s1, File S1: Prior information, models and model results. File S2: Study protocol. File S3: STARD checklist.
