# Supplementary material for: Evaluation of an Antibody Detecting Point of Care Test for Diagnosis of Taenia solium Cysticercosis in a Zambian Rural Community: A Prospective Diagnostic Accuracy Study
Source: Diagnostics (Basel). 2021 Nov 15;11(11):2121. doi: 10.3390/diagnostics11112121 (PMC8618153; doi:10.3390/diagnostics11112121)
Supplement: Supplementary file 1 [file diagnostics-11-02121-s001.zip › cysticercosis diagnosis/File S1.pdf]

File S1

Prior information

Probabilistic constraints used were in form of expert opinion obtained from three experts from the Institute of Tropical Medicine (PD), Ghent University (SG) and the University of Zambia (KEM) (S1 File).

Table S1. Expert prior information on prevalence, sensitivity and specificity. The analysis allows the sensitivities and specificities to differ conditional on the other test-results.

Test	Lower Limit	Upper Limit
Prevalence	0.05	0.3
LLGP EITB - sensitivity	0.8	0.99
LLGP EITB - specificity	0.7	0.99
rT24H EITB - sensitivity	0.9	0.99
rT24H EITB - specificity	0.9	0.99
Serum Ag ELISA - sensitivity	0.5	0.99
Serum Ag ELISA - specificity	0.9	0.99

Models TS POC CC-rT24H EITB-serum Ag ELISA

Table S2. Models used for TS POC CC analysis without LLGP EITB

Parameters	Model1	Model2	Model3
theta1	[0.05;0.3]	[0.05;0.3]	[0.05;0.3]
theta2	[0;1]	[0;1]	[0;1]
theta3	[0.5,1]	[0.5,1]	[0.5,1]
theta4	[0;1]	[0.9;0.99]	[0.9;0.99]
theta5	[0;1]	[0.9;0.99]	[0.9;0.99]
theta6	[0.5,1]	[0.9;0.99]	[0.9;0.99]
theta7	[0.5,1]	[0.9;0.99]	[0.9;0.99]
theta8	[0;1]	[0;1]	[0.5;0.99]

theta9	[0;1]	[0;1]	[0.5;0.99]
theta10	[0;1]	[0;1]	[0.5;0.99]
theta11	[0;1]	[0;1]	[0.5;0.99]
theta12	[0.5,1]	[0.5,1]	[0.9;0.99]
theta13	[0.5,1]	[0.5,1]	[0.9;0.99]
theta14	[0.5,1]	[0.5,1]	[0.9;0.99]
theta15	[0.5,1]	[0.5,1]	[0.9;0.99]

Models including LLGP EITB

Table S3. Models used for TS POC CC analysis with all tests. The analysis was repeated with prior information on prevalence (theta 1) [0; 0.3] instead.

Parameters	Model1	Model2	Model3	Model4
theta1	[0.05;0.3]	[0.05;0.3]	[0.05;0.3]	[0.05;0.3]
theta2	[0;1]	[0;1]	[0;1]	[0;1]
theta3	[0.5,1]	[0.5,1]	[0.5,1]	[0.5,1]
theta4	[0;1]	[0.8;0.99]	[0.8;0.99]	[0.8;0.99]
theta5	[0;1]	[0.8;0.99]	[0.8;0.99]	[0.8;0.99]
theta6	[0.5,1]	[0.7;0.99]	[0.7;0.99]	[0.7;0.99]
theta7	[0.5,1]	[0.7;0.99]	[0.7;0.99]	[0.7;0.99]
theta8	[0;1]	[0;1]	[0.9;0.99]	[0.9;0.99]
theta9	[0;1]	[0;1]	[0.9;0.99]	[0.9;0.99]
theta10	[0;1]	[0;1]	[0.9;0.99]	[0.9;0.99]
theta11	[0;1]	[0;1]	[0.9;0.99]	[0.9;0.99]
theta12	[0.5,1]	[0.5,1]	[0.9;0.99]	[0.9;0.99]
theta13	[0.5,1]	[0.5,1]	[0.9;0.99]	[0.9;0.99]
theta14	[0.5,1]	[0.5,1]	[0.9;0.99]	[0.9;0.99]
theta15	[0.5,1]	[0.5,1]	[0.9;0.99]	[0.9;0.99]
theta16	[0;1]	[0;1]	[0;1]	[0.5;0.99]
theta17	[0;1]	[0;1]	[0;1]	[0.5;0.99]
theta18	[0;1]	[0;1]	[0;1]	[0.5;0.99]
theta19	[0;1]	[0;1]	[0;1]	[0.5;0.99]
theta20	[0;1]	[0;1]	[0;1]	[0.5;0.99]

theta21	[0;1]	[0;1]	[0;1]	[0.5;0.99]
theta22	[0;1]	[0;1]	[0;1]	[0.5;0.99]
theta23	[0;1]	[0;1]	[0;1]	[0.5;0.99]
theta24	[0.5,1]	[0.5,1]	[0.5,1]	[0.9;0.99]
theta25	[0.5,1]	[0.5,1]	[0.5,1]	[0.9;0.99]
theta26	[0.5,1]	[0.5,1]	[0.5,1]	[0.9;0.99]
theta27	[0.5,1]	[0.5,1]	[0.5,1]	[0.9;0.99]
theta28	[0.5,1]	[0.5,1]	[0.5,1]	[0.9;0.99]
theta29	[0.5,1]	[0.5,1]	[0.5,1]	[0.9;0.99]
theta30	[0.5,1]	[0.5,1]	[0.5,1]	[0.9;0.99]
theta31	[0.5,1]	[0.5,1]	[0.5,1]	[0.9;0.99]

Table S4. Diagnostic characteristics of tests when LLGP EITB results are included, with a cysticercosis prevalence near zero.

Parameter	TS POC CC	LLGP EITB	rT24H EITB	Serum Ag ELISA
Sensitivity %	72 (30-99)	89 (81-97)	72 (41-93)	63 (38-85)
Specificity %	86 (83-89)	98 (96-99)	90 (86-94)	86 (81-91)
PPV %	9 (3-17)	44 (17-68)	13 (4-26)	8 (3-17)
NPV %	99 (97-100)	100 (99-100)	100 (98-100)	99 (98-100)

Prevalence 2% (0.1-4%), Bayesian P 0.55. Abbreviations: TS POC CC= *Taenia solium* point of care test for cysticercosis; LLGP EITB= Lentil lectin glycoprotein enzyme linked immunoelectrotransfer blot; rT24H EITB= recombinant T24H glycoprotein enzyme-linked immunoelectrotransfer blot; Serum Ag ELISA= serum antigen enzyme linked immunosorbent assay
