# Supplementary material for: Evaluation of an Antibody Detecting Point of Care Test for Diagnosis of Taenia solium Cysticercosis in a Zambian Rural Community: A Prospective Diagnostic Accuracy Study
Source: Diagnostics (Basel). 2021 Nov 15;11(11):2121. doi: 10.3390/diagnostics11112121 (PMC8618153; doi:10.3390/diagnostics11112121)
Supplement: Supplementary file 1 [file diagnostics-11-02121-s001.zip › cysticercosis diagnosis/S3 File_STARD checklist 27092021.pdf]

File S3. STARD checklist

Section & Topic	No	Item	Section (page)	Explanation
TITLE OR ABSTRACT				
	1	Identification as a study of diagnostic accuracy using at least one measure of accuracy (such as sensitivity, specificity, predictive values, or AUC)	Title (1)<br>Abstract (2)<br>Keywords (4)	The subtitle indicates the study: "A prospective diagnostic accuracy study"<br>Keywords: "sensitivity", "specificity"
ABSTRACT				
	2	Structured summary of study design, methods, results, and conclusions<br>(for specific guidance, see STARD for Abstracts)	Abstract (2)	The abstract was written according to the STARD guidelines for abstracts
INTRODUCTION				
	3	Scientific and clinical background, including the intended use and clinical role of the index test	Introduction (5)	Intended use: surveillance and follow up of control interventions<br>Clinical role: new test: The lack of cheap, easy-to-use, rapid diagnostic tests has led to the development of several rapid diagnostic tests for cysticercosis. Despite this, none of the developed tests have been evaluated for practical use in either health facilities or communities
	4	Study objectives and hypotheses	Methods	The primary objective of this paper was to assess the sensitivity and specificity of the TS POC CC test strip (index test) for the detection of HCC in *T. solium* endemic communities in Zambia.
METHODS				
Study design	5	Whether data collection was planned before the index test and reference standard were performed (prospective study) or after (retrospective study)	Study setting, design and participant recruitment (7)	Prospective study: participants were recruited from randomly selected households

Participants	6	Eligibility criteria	Study setting, design and participant recruitment (7)	To participate in the study, participants had to live in the study area, be 10 years of age or older, give written informed consent and express willingness to participate in all study aspects such as, getting tested, provision of blood and stool samples, as well as going for CT scan if and when it was required. Those who were reported severely ill, pregnant or visiting the study area were not recruited for the study.
	7	On what basis potentially eligible participants were identified (such as symptoms, results from previous tests, inclusion in registry)	Study setting, design and participant recruitment (7)	Study participants were recruited from randomly selected households. The eligibility criteria were not strict, in order to obtain a representative sample from the population.
	8	Where and when potentially eligible participants were identified (setting, location and dates)	Study setting, design and participant recruitment (7)	Recruitment was done in four communities in Sinda district, Eastern Province of Zambia. The recruitment took place from December 2017 to June 2019.
	9	Whether participants formed a consecutive, random or convenience series	Study setting, design and participant recruitment (7)	Households were randomly selected and all consenting eligible individuals in participating households were recruited.
Test methods	10a	Index test, in sufficient detail to allow replication	Index test - the TS POC CC test strip (8)	TS POC CC test strip: a lateral flow assay using the recombinant protein rT24H.
	10b	Reference standard, in sufficient detail to allow replication	Reference tests (10)	Three reference tests were used, the LLGP EITB [4], the rT24H EITB [7,17], which has a performance concordant to the LLGP EITB [8] and the B158/60 serum Ag ELISA [9].
	11	Rationale for choosing the reference standard (if alternatives exist)	Introduction	LLGP EITB is very sensitive and specific and the test of choice for cysticercosis serodiagnosis [3–6]. A recombinant based EITB using

				the rT24H antigen has been established (rT24H EITB) [7]. Two antigen detecting ELISAs, the B158/60 (serum Ag ELISA) [9] and the HP10 antigen ELISA [10] are available and recommended for use in diagnosis [11,12].
	12a	Definition of and rationale for test positivity cut-offs or result categories<br>of the index test, distinguishing pre-specified from exploratory	Index test - the TS POC CC test strip (8)	A pink line at the test mark indicated a positive result and its absence a negative one.
	12b	Definition of and rationale for test positivity cut-offs or result categories<br>of the reference standard, distinguishing pre-specified from exploratory	Reference tests (10)	Pre-specified cut-offs were used for serum Ag ELISA. The EITB tests were positive/negative (visible test line or not).
	13a	Whether clinical information and reference standard results were available<br>to the performers/readers of the index test	NA	The index test was performed before the reference tests. People were recruited from the community.
	13b	Whether clinical information and index test results were available<br>to the assessors of the reference standard	Reference tests (10)	One researcher (CM) who was part of the recruitment in the community also took part in the laboratory analysis in Zambia. The other laboratory analysts were completely blinded to the TS POC result and the serum Ag ELISA or EITB results. The EITB tests were performed in parallel (so not blinded to the other EITB results).
Analysis	14	Methods for estimating or comparing measures of diagnostic accuracy	Data analysis (11)	A Bayesian analysis was used.
	15	How indeterminate index test or reference standard results were handled	Data analysis (11); Fig 1	Indeterminate index test results were excluded from the primary analysis

	16	How missing data on the index test and reference standard were handled	Data analysis (11); Fig 1	Only complete cases were used for the primary analysis
	17	Any analyses of variability in diagnostic accuracy, distinguishing pre-specified from exploratory	Data analysis (11)	N/A (subgroup analyses were not performed in this study) Agreement between tests was an exploratory outcome of the study
	18	Intended sample size and how it was determined	Study setting, design and participant recruitment (7)	The sample size was set at 1200 participants, in order to obtain a precision of sensitivity and specificity of 10%
RESULTS				
Participants	19	Flow of participants, using a diagram	Fig 2	Extra information is given in "Flow and demographic characteristics of participants" (p14)
	20	Baseline demographic and clinical characteristics of participants	Flow and demographic characteristics of participants (15)	The median age of the participants was 28 years (range 10-95 years) and 57.3% were female. No clinical characteristics were recorded for the evaluation of the TS POC CC test strip; Participants were recruited from the community.
	21a	Distribution of severity of disease in those with the target condition	NA	Participants were recruited from the general population. There is no gold standard to diagnose HCC.
	21b	Distribution of alternative diagnoses in those without the target condition	NA	Participants were recruited from the general population. There is no gold standard to diagnose HCC.
	22	Time interval and any clinical interventions between index test and reference standard	Sampling and sample processing (p8)	Samples were taken immediately after the TS POC test
Test results	23	Cross tabulation of the index test results (or their distribution) by the results of the reference standard	Figure 2, Table 2	
	24	Estimates of diagnostic accuracy and their precision	Table 3	The analysis resulted in an estimated sensitivity and

		(such as 95% confidence intervals)		specificity of 35% (95% CI: 14-63%) and 87% (95% CI: 83-90%) for the TS POC CC test
	25	Any adverse events from performing the index test or the reference standard	Index test - the TS POC CC test strip (8)	There were no adverse events recorded (only a finger prick, blood and stool collection was performed)
DISCUSSION				
	26	Study limitations, including sources of potential bias, statistical uncertainty, and generalisability	Discussion	The results of this study may not be generalized to communities with a different disease spectrum. Potential biases could occur from the high number of sampling refusals especially on TS POC double negative participants in case they would have a different disease spectrum compared to the participating population.
	27	Implications for practice, including the intended use and clinical role of the index test	Discussion	The TS POC CC test in its current form cannot be used in practice due to the low sensitivity and specificity
OTHER INFORMATION				
	28	Registration number and name of registry	Trial registration (12)	The SOLID project was registered under the Pan African Clinical Trial Registry PACTR201712002788889
	29	Where the full study protocol can be accessed	S2 file	The protocol is included as supporting information of this manuscript. A study design paper has been submitted to Diagnostics Journal, therefore the protocol has only be included for review at this stage
	30	Sources of funding and other support; role of funders	Financial disclosure section of the submission system	This work was funded by the European & Developing Countries Clinical Trials Partnership (EDCTP; grant number DRIA2014-308 SOLID) and the German Federal Ministry of Education and Research (BMBF; grant number:

				01KA1617) within the research grant “Evaluation of an antibody detecting point-of-care test for the diagnosis of *Taenia solium* taeniosis and (neuro)cysticercosis in communities and primary care settings of highly endemic, resource-poor areas in Tanzania and Zambia, including training of – and technology transfer to the Regional Reference Laboratory and health centers (SOLID)”. The funders had no role in this study.
